# Myosteatosis as a novel prognostic biomarker after radical cystectomy for bladder cancer

**DOI:** 10.1038/s41598-020-79340-9

**Published:** 2020-12-17

**Authors:** Shimpei Yamashita, Yuya Iwahashi, Haruka Miyai, Takashi Iguchi, Hiroyuki Koike, Satoshi Nishizawa, Nagahide Matsumura, Keizo Hagino, Kazuro Kikkawa, Yasuo Kohjimoto, Isao Hara

**Affiliations:** 1 Department of Urology, Wakayama Medical University, 811-1 Kimiidera, Wakayama, 641-0012 Japan; 2 Department of Urology, Kinan Hospital, Wakayama, Japan; 3 Department of Urology, Rinku General Medical Center, Osaka, Japan; 4 Department of Urology, Naga Municipal Hospital, Wakayama, Japan

**Keywords:** Cancer, Oncology, Urology

## Abstract

This study aims to evaluate the influence of myosteatosis on survival of patients after radical cystectomy (RC) for bladder cancer. We retrospectively identified 230 patients who underwent RC for bladder cancer at our three institutions between 2009 and 2018. Digitized free-hand outlines of the left and right psoas muscles were made on axial non-contrast computed tomography images at level L3. To assess myosteatosis, average total psoas density (ATPD) in Hounsfield Units (HU) was also calculated as an average of bilateral psoas muscle density. We compared cancer-specific survival (CSS) between high ATPD and low ATPD groups and performed cox regression hazard analyses to identify the predictors of CSS. Median ATPD was 44 HU (quartile: 39–47 Hounsfield Units). Two-year CSS rate in overall patients was 76.6%. Patients with low ATPD (< 44 HU) had significantly lower CSS rate (*P* = 0.01) than patients with high ATPD (≥ 44 HU). According to multivariate analysis, significant independent predictors of poor CSS were: Eastern Cooperative Oncology Group performance status ≥ 1 (*P* = 0.03), decreasing ATPD (*P* = 0.03), non-urothelial carcinoma (*P* = 0.01), pT ≥ 3 (*P* < 0.01), and pN positive (*P* < 0.01). In conclusion, myosteatosis (low ATPD) could be a novel predictor of prognosis after RC for bladder cancer.

## Introduction

Radical cystectomy (RC) is the standard treatment for patients with muscle-invasive bladder cancer, patients with high-risk non-muscle invasive bladder cancer, and patients with carcinoma in situ (CIS) resistant to bacillus Calmette-Guerin (BCG) treatment^1–3^. Meanwhile, five-year overall survival (OS) rates are 42–58%, despite RC^4,5^. Depending on the patient’s condition, bladder-preserving therapy by combined modality therapy could be a treatment option^6^. To select optimal management on an individual basis, it is therefore important to identify preoperative prognostic factors for patients who undergo RC.

Sarcopenia, defined as severe wasting of skeletal muscle mass, has been reported in several recent studies to be a preoperative prognostic factor in patients who undergo RC for bladder cancer, and is associated with poor rates of survival after RC^7–9^. Meanwhile, myosteatosis has recently drawn attention as a novel and objective preoperative prognostic factor in patients with various cancers, including gastric cancer, colorectal cancer, pancreatic cancer, lung cancer and ovarian cancer^10–14^. Myosteatosis is defined as increased fat infiltration in skeletal muscle^15^. Sarcopenia concerns muscle quantity and can be evaluated on abdominal computed tomography (CT) by the volume of skeletal muscle or psoas muscle^7–9^. Myosteatosis, by contrast, concerns muscle quality and can be evaluated on abdominal CT by the CT attenuation value of skeletal muscle or psoas muscle^10–14^.

To our knowledge, the association between myosteatosis and survival after RC for bladder cancer has not been investigated. We hypothesized that myosteatosis could be a novel preoperative prognostic factor in patients who undergo RC. In the present study, we evaluate the association between various parameters, including sarcopenia and myosteatosis, and survival after RC in patients with bladder cancer. To assess sarcopenia and myosteatosis, we measured psoas muscle index (PMI) and average total psoas density (ATPD), respectively.

## Results

Patient demographics are summarized in Table 1. Median age was 73 years (quartile: 67–79 years) and 184 patients were male (80%). Median PMI and ATPD were 4.6 cm^2^/m^2^ (quartile: 3.6–5.6 cm^2^/m^2^) and 44 HU (quartile: 39–47 HU), respectively. Sixty-five patients (28%) received neoadjuvant chemotherapy. There were concerns about the possible potential impact of neoadjuvant chemotherapy on the myosteatosis measurements, but in these patients, median ATPD difference before and after neoadjuvant chemotherapy was 0 HU (quartile: − 2.5–1.0 HU) and there was no notable change in ATPD before and after neoadjuvant chemotherapy. Most patients underwent open RC (82%). Pathological diagnosis was urothelial carcinoma (UC) in 209 patients (91%) and non-UC in 21 patients (9%, squamous cell carcinoma in 10 patients, small cell carcinoma in 7 patients and other histopathological type in 4 patients). Pathological T stage of resected specimen was ≤ pT2 in 142 patients (62%) and ≥ pT3 in 88 patients (38%). Pathological lymph node metastasis was observed in 41 patients (18%).

**Table 1:** Patient demographics.

Age, years	73 (67–79)
Male, n (%)	184 (80)
BMI, kg/m^2^	22.2 (19.8–24.2)
ECOG PS ≥ 1, n (%)	40 (17)
CCI ≥ 1, n (%)	105 (46)
Neutrophil-to-lymphocyte ratio	2.2 (1.6–3,2)
Serum albumin, g/dL	4.0 (3.3–4.3)
Psoas muscle index, cm^2^/m^2^	4.6 (3.6–5.6)
Average psoas muscle density, HU	44 (39–47)
Neoadjuvant chemotherapy, n (%)	65 (28)
Cystectomy approach, n (%)	
*Open*	189 (82)
*Laparoscopic*	23 (10)
*Robotic*	18 (8)
Urinary diversion, n (%)	
Cutaneous ureterostomy	91 (40)
Ileal conduit	118 (51)
Neobladder	21 (9)
Pathological diagnosis, n (%)	
*UC*	209 (91)
*Non-UC*	21 (9)
Pathological T stage, n (%)	
pT0–T2	142 (62)
pT3–T4	88 (38)
Lymph node metastasis	
pN negative	189 (82)
pN positive	41 (18)
CIS concurrent	
*Yes*	49 (21)
*No*	181(79)

*Continuous variables are shown in “median (quartile)” form.

**Figure 1:**
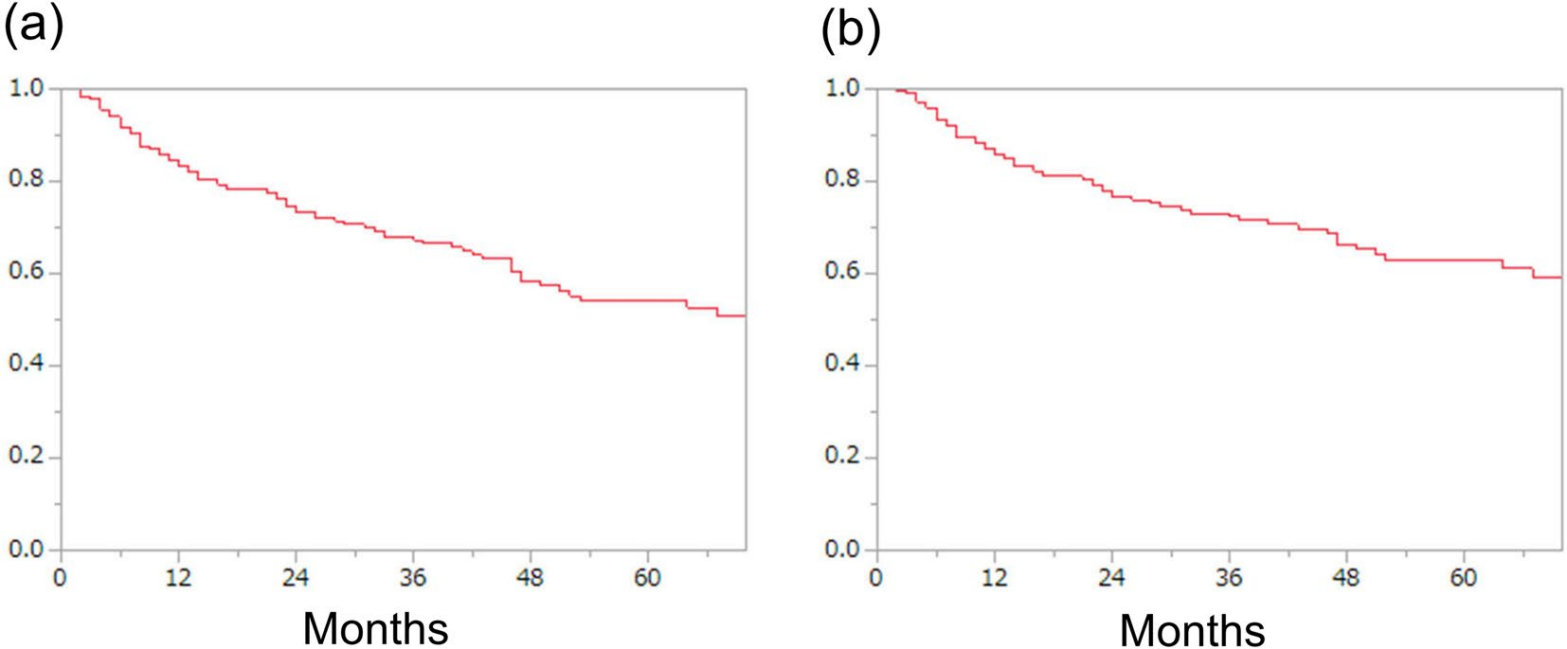
Kaplan–Meier plots of (**a**) overall survival and (**b**) cancer-specific survival.

**Figure 2:**
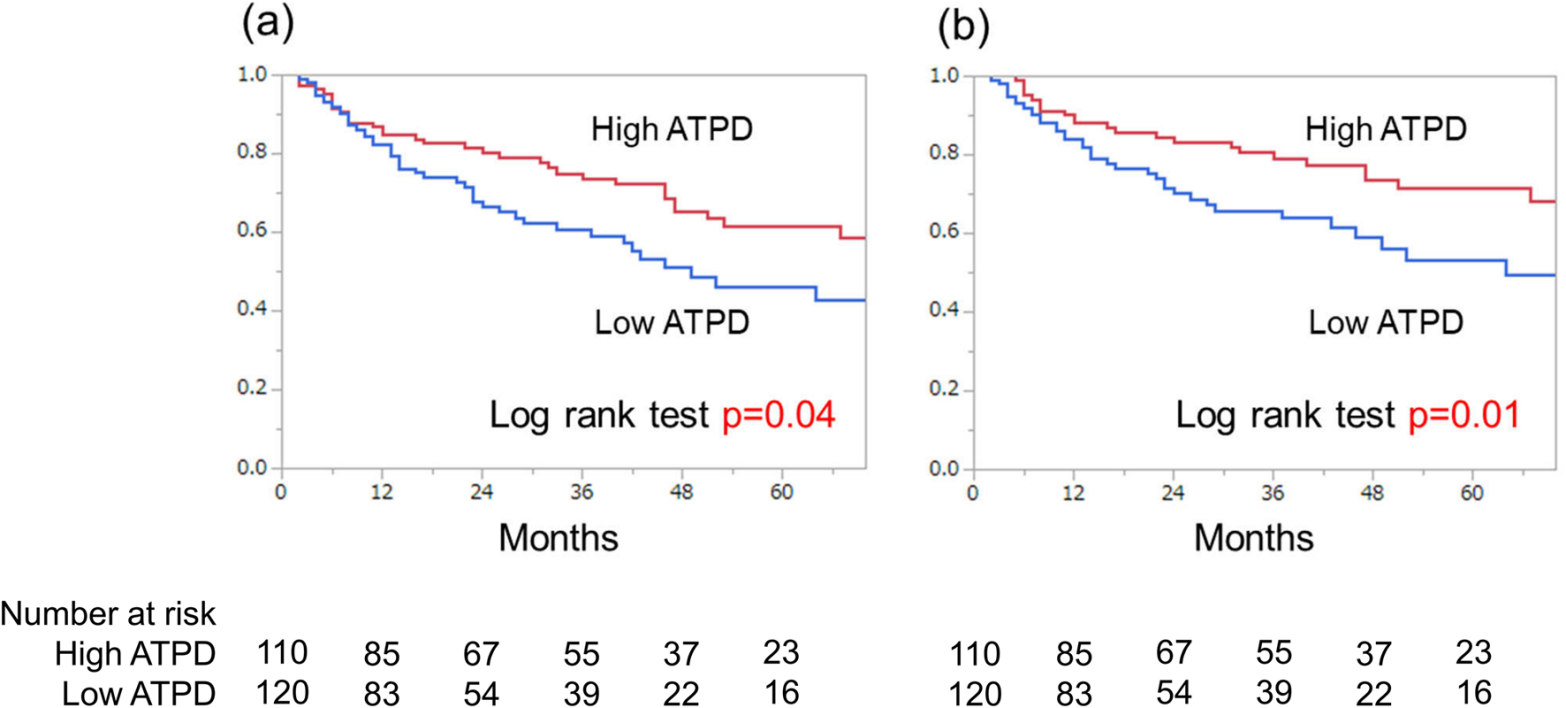
Comparison of (**a**) overall survival and (**b**) cancer-specific survival between patients with high average total psoas density (≥ 44 HU) and low average total psoas density (< 44 HU).

During the observation period (median 25.5 months, quartile: 10.8–49.3 months), 62 patients died of bladder cancer (27%) and 18 patients died of another cause (8%). The two-year OS rate and two-year cancer specific survival (CSS) rate were 73.4% and 76.6%, respectively (Fig. 1). We classified the patients into high ATPD (≥ 44 HU) and low ATPD (< 44 HU) groups using the median ATPD as cutoff value, and we compared OS and CSS between the two groups. The patients with low ATPD had significantly lower rate of OS (*P* = 0.04) and lower rate of CSS (*P* = 0.01) than patients with high ATPD (Fig. 2). Patient demographics are compared in Table 2. The patients in the low ATPD group were significantly older and had lower ratio of males, higher body mass index (BMI), higher percentage of poor Eastern Cooperative Oncology Group performance status (ECOG PS) and lower psoas muscle index than those in the high ATPD group.

**Table 2:** Comparison of patient demographics between patients with high ATPD (≥ 44 HU) and those with low ATPD (< 44 HU).

	High ATPD	Low ATPD	*P* value
No. pts	110	120	
Age, years	71 (63–77)	75 (70–79)	< 0.01
Male, n (%)	99 (90)	85 (71)	< 0.01
BMI, kg/m^2^	21.5 (18.8–23.6)	22.7 (20.7–25.2)	< 0.01
ECOG PS ≥ 1, n (%)	12 (11)	28 (23)	0.01
CCI ≥ 1, n (%)	46 (42)	59 (50)	0.23
Neutrophil-to-lymphocyte ratio	2.1 (1.5–3.3)	2.2 (1.6–3.0)	0.86
Serum albumin, g/dL	4.0 (3.6–4.3)	3.9 (3.6–4.2)	0.17
Psoas muscle index, cm^2^/m^2^	4.9 (3.8–5.7)	4.2 (3.3–5.4)	0.01
Neoadjuvant chemotherapy, n (%)	27 (25)	38 (32)	0.22
Cystectomy approach, n (%)			0.49
*Open*	88 (80)	101 (84)	
*Laparoscopic*	11 (10)	12 (10)	
*Robotic*	11 (10)	7 (6)	
Urinary diversion, n (%)			< 0.01
*Cutaneous ureterostomy*	39 (35)	52 (43)	
*Ileal conduit*	54 (49)	64 (53)	
*Neobladder*	17 (15)	4 (3)	
Pathological diagnosis, n (%)			0.98
*UC*	100 (91)	109 (91)	
*Non-UC*	10 (9)	11 (9)	
Pathological T stage, n (%)			0.16
*pT0-T2*	73 (66)	69 (58)	
*pT3-T4*	37 (34)	51 (43)	
Lymph node metastasis			0.89
*pN negative*	90 (82)	99 (83)	
*pN positive*	20 (18)	21 (18)	
CIS concurrent			0.14
*Yes*	28 (25)	21 (18)	
*No*	82 (75)	99 (83)	

*Continuous variables are shown in “median (quartile)” form.

Table 3 shows the results of univariate and multivariate cox proportional analyses of associations between various parameters and OS. In univariate analysis, the following were significantly associated with poor OS: older age (*P* = 0.01), ECOG PS ≥ 1 (*P* < 0.01), Charlson Comorbidity Index (CCI) ≥ 1 (*P* = 0.04), low PMI (*P* = 0.02), low ATPD (*P* = 0.01), non UC (*P* < 0.01), pT ≥ 3 (*P* < 0.01) and pN positivity(*P* < 0.01). Multivariate analysis showed that ECOG PS ≥ 1 (*P* = 0.03), pT ≥ 3 (*P* < 0.01) and pN positive (*P* < 0.01) were significant independent predictors of poor OS. Moreover, increasing age was a marginally significant predictive factor of OS (*P* = 0.06). On the other hand, PMI (*P* = 0.11) and ATPD (*P* = 0.18) were not independently significant.

**Table 3:** Univariate and multivariate analyses of associations between various parameters and overall survival.

Variable	Univariate analysis			Multivariate analysis		
	HR	95% CI	*P* value	HR	95% CI	*P* value
Age	1.03	1.00–1.06	0.01	1.03	0.99–1.06	0.06
Male	0.98	0.56–1.70	0.95			
≥ ECOG PS 1	2.74	1.61–4.65	< 0.01	1.91	1.04–3.49	0.03
CCI 1 or more	1.59	1.02–2.48	0.04	1.25	0.79–1.98	0.33
Psoas muscle index	0.82	0.69–0.96	0.02	0.87	0.73–1.03	0.11
Average psoas muscle density	0.96	0.94–0.99	0.01	0.98	0.95–1.00	0.18
Neoadjuvant chemotherapy	0.97	0.59–1.59	0.90			
non UC (vs UC)	2.77	1.48–5.18	< 0.01	1.72	0.89–3.34	0.10
≥ pT3	3.63	2.31–5.71	< 0.01	2.72	1.65–4.48	< 0.01
pN positivity	2.29	1.38–3.79	< 0.01	2.27	1.30–3.96	< 0.01
Concurrent CIS	0.85	0.48–1.49	0.58			

Table 4 shows the results of univariate and multivariate cox proportional analyses of associations between various parameters and CSS. According to univariate analysis, the following were significantly associated with poor CSS: ECOG PS ≥ 1 (*P* = 0.01), low ATPD (*P* < 0.01), non-UC (*P* < 0.01), pT ≥ 3 (*P* < 0.01), and pN positivity (*P* < 0.01). Meanwhile, significant independent predictors of poor CSS according to multivariate analysis were: ECOG PS ≥ 1 (*P* = 0.03), low ATPD (*P* = 0.03), non-UC (*P* = 0.01), pT ≥ 3 (*P* < 0.01), and pN positivity (*P* < 0.01).

**Table 4:** Univariate and multivariate analyses of associations between various parameters and cancer-specific survival.

Variable	Univariate analysis			Multivariate analysis		
	HR	95% CI	*P* value	HR	95% CI	*P* value
Age	1.00	0.97–1.04	0.58			
Male	0.86	0.47–1.57	0.64			
≥ ECOG PS 1	2.20	1.18–4.10	0.01	2.05	1.04–4.04	0.03
≥ CCI 1	1.23	0.75–2.03	0.40			
Psoas muscle index	0.87	0.72–1.04	0.13			
Average psoas muscle density	0.96	0.93–0.99	< 0.01	0.96	0.94–0.99	0.03
Neoadjuvant chemotherapy	0.95	0.54–1.65	0.85			
non-UC (vs. UC)	3.60	1.90–6.83	< 0.01	2.26	1.15–4.43	0.01
≥ pT3	4.40	2.60–7.43	< 0.01	2.98	1.68–5.27	< 0.01
pN positivity	2.75	1.60–4.73	< 0.01	2.26	1.25–4.08	< 0.01
Concurrent CIS	0.69	0.35–1.37	0.29			

**Figure 3:**
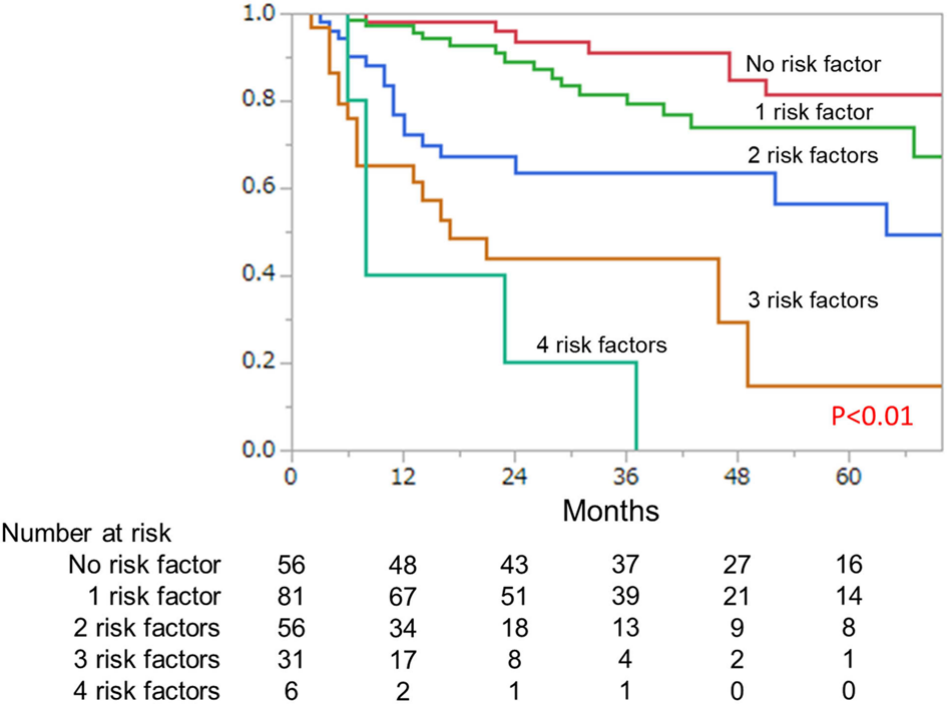
Kaplan–Meier curves for cancer-specific survival according to risk group classification.

To develop a risk classification to predict CSS after radical cystectomy in patients with bladder cancer, five risk factors, (ECOG PS ≥ 1, ATPD < 44HU, non-UC, pT ≥ 3, and pN positivity) were used, and the cohort was classified into five groups according to the presence of these five risk factors. This model effectively stratified patients in terms of CSS according to the number of risk factors (*P* < 0.01), as shown in Fig. 3.

Table 5 shows the results of comparison of OS and CSS between high ATPD and low ATPD groups by using various cutoff values of ATPD. When using 35HU and 44HU (median value in the present study) as cutoff values, OS and CSS rates in low ATPD group were significantly lower than those in high ATPD group. On the other hand, when using other values, there was no statistically significant difference between two groups in OS and CSS rates.

**Table 5:** Comparison of overall survival and cancer-specific survival between high ATPD and low ATPD groups by using various cutoff values of ATPD.

Cutoff value (HU)	Two-year OS rate		*P* value (log rank test)	Two-year CSS rate		*P* value (log rank test)
	High ATPD	Low ATPD		High ATPD	Low ATPD	
35	77.0% (n = 199)	51.7% (n = 31)	< 0.01	79.9% (m = 199)	56.7% (n = 31)	< 0.01
40	76.8% (n = 163)	65.1% (n = 67)	0.19	80.3% (n = 163)	67.7% (n = 67)	0.05
44 (present study)	80.2% (n = 110)	66.4% (n = 120)	0.04	83.2% (n = 110)	70.1% (n = 120)	0.01
50	84.6% (n = 26)	71.7% (n = 204)	0.14	84.6% (n = 26)	75.4% (n = 204)	0.33

## Discussion

We examined low ATPD, namely myosteatosis, as a possible preoperative predictor of prognosis after RC in patients with bladder cancer. To the best of our knowledge, this is the first report about the clinical significance of myosteatosis for predicting prognosis after RC. Patients with low ATPD had lower OS and CSS rates after RC than those with high ATPD. Low ATPD was a significant independent predictor of poor CSS in patients who underwent RC for their bladder cancer.

Muscle depletion has recently drawn attention as a prognostic factor in patients with various forms of cancer. It is classified into reduced muscle volume (sarcopenia) and declined muscle quality (myosteatosis)^15,16^ and can occur in any weight category, from underweight to obese^10,17,18^. Sarcopenia has been reported to be associated with prognosis for patients with various forms of cancer, including bladder cancer^9^. In addition, myosteatosis has been shown to be a novel predictive factor in patients with other types of cancer, such as gastric cancer, colorectal cancer, pancreatic cancer, ovarian cancer and breast cancer^10–12,14,19^. Little is known, however, about the association between myosteatosis and the prognosis of patients with bladder cancer.

Sarcopenia has been evaluated by measuring the area or volume of skeletal muscle or psoas muscle on CT images^7–9^. Myosteatosis, meanwhile, has been defined as decreased muscle attenuation values and evaluated by measuring the CT attenuation values of skeletal muscles or psoas muscles^10,20^. In this study we measured ATPD on the CT image at level L3, and examined the association between ATPD and the prognosis after RC in patients with bladder cancer. Patients with low ATPD had poorer OS and CSS than those with high ATPD. Moreover, low ATPD was an independent significant predictor of CSS after RCC in patients with bladder cancer. These results suggest that myosteatosis could be a novel predictive factor of poor prognosis after RC in patients with bladder cancer.

We developed a risk classification model based on various parameters, including low ATPD for patients who undergo RC. To our knowledge, this is the first study to establish a risk classification or nomogram prediction of CSS based on prognostic parameters including myosteatosis in patients with bladder cancer undergoing RC. We believe that our risk classification will be helpful in predicting prognosis after RC in patients with bladder cancer.

**Figure 4:**
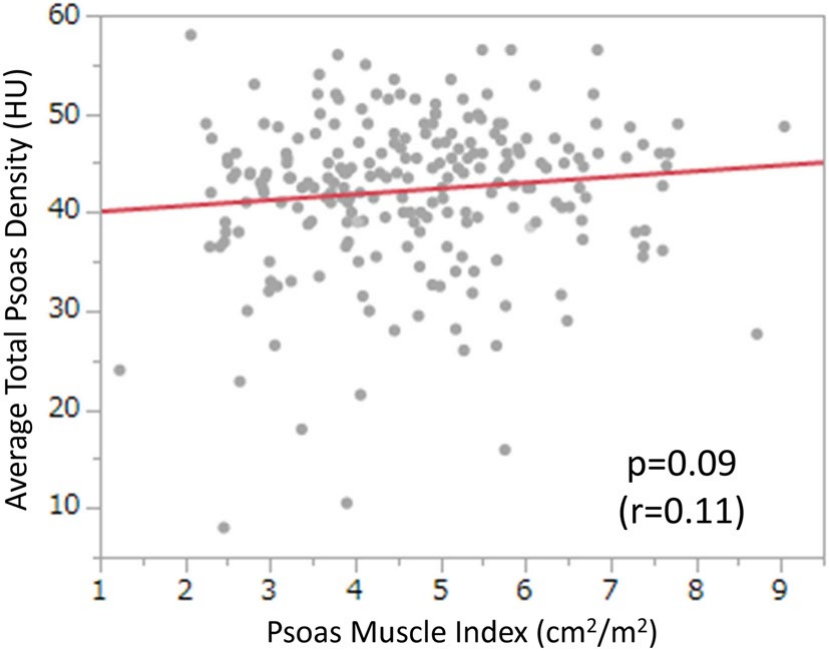
Scatterplot of the relationship between psoas muscle index and average total psoas density.

The prognostic impact of sarcopenia is thought to be due to a combination of vulnerability to cancer and its treatments, due to low physical reserves, or to sub-optimal treatment options in patients with limited physical reserves^19,21^. Meanwhile, the reason for myosteatosis leading to poor prognosis in patients with malignant diseases, remains unclear. To examine the association between sarcopenia and myosteatosis, we investigated the relationship between PMI and ATPD, but there was no significant correlation (Fig. 4, Spearman’s rank correlation coefficient 0.11, *P* = 0.09). This suggested that myosteatosis worsened the prognosis after RC in patients with bladder cancer by a mechanism different to sarcopenia. Several possible mechanisms have been previously suggested. Skeletal muscle is known to be secretory and muscle cells secrete cytokines and other peptides, which may influence the growth and metastasis of tumor cells^10,22^. Reduced muscle quality by myosteatosis can therefore lead to an altered myokine response and deficient regulation of tumor cells. Moreover, myosteatosis is associated with hyperinsulinemia and insulin resistance^23,24^. Hyperinsulinemia can promote tumor cell proliferation through insulin receptor^25^. The decline in synthesis of insulin-like growth factor-1 (IGF-1) binding protein and activation of IGF-1 by hyperinsulinemia can also lead to tumor cell proliferation^25^. Furthermore, myosteatosis promotes an elevated systemic inflammatory response. Inflammation stimulates tumor cell proliferation and can lead to poorer chance of cancer survival^26–28^. Further studies will seek to clarify how myosteatosis influences the prognosis of patients with bladder cancer.

The current study has several limitations. It was a retrospective study and the results require verification by a large-scale prospective study. The timing of preoperative CT scans was also inconsistent, although only patients with preoperative CT examination within 30 days of RC were included in the present study. To perform large-scale prospective studies, it is therefore necessary to recruit a large number of patients who will undergo radical cystectomy for their bladder cancer and to standardize the timing of preoperative CT scans and post-operative follow-up protocol. Moreover, although we used median ATPD (44 HU) as cutoff value, there is no consensus about optimal cutoff value of ATPD or skeletal muscle density. Interestingly, the used cutoff values of muscle attenuation value for evaluating myosteatosis status differ among previous studies. Alexio et al. used 37.8 HU as cutoff value in their studies of patients with breast cancer^19^, while Aust et al. used 39 HU for ovarian cancer^14^. Martin et al. adopted 33–41 HU according to BMI in a cohort with various cancers^13^. Cutoff value was decided in some studies by gender (male: 35.5–38.8 HU, female: 28.6–32.5 HU)^10,11^. As shown in Table 5, the results of comparison of OS and CSS between high ATPD and low ATPD differ depending on the cutoff value used. In Cox proportional analyses, however, ATPD was analyzed as a continuous variable and, as a result, poor prognostic impact of decreasing ATPD was shown. We therefore believe that myosteatosis is associated with poor prognosis in patients that have undergone radical cystectomy. Further consideration will be required to decide the optimal cutoff value of ATPD to define myosteatosis.

In conclusion, myosteatosis (low ATPD) was indicated to be independently associated with poor CSS in our patients who underwent RC for bladder cancer. The development of risk classifications or nomograms with inclusion of myosteatosis may be clinically useful for patients with bladder cancer.

## Methods

### Patient selection

We retrospectively reviewed the records of consecutive patients who underwent RC for bladder cancer at the Wakayama Medical University Hospital, Kinan Hospital and Rinku General Medical Center between March 2009 and March 2018. Patients were excluded from this study if preoperative abdominal CT examination was not available within 30 days of surgery, or if they did not receive post-operative follow-up at one of the institutions. Of 239 candidates, 230 patients were finally enrolled in the study. This multi-institutional retrospective study was approved by the Wakayama Medical University Institutional Review Board (approval number 3008) and conducted in accordance with the principles of the Declaration of Helsinki. All participants gave written informed consent prior to the study.

### Data collection

Patient demographic data at operation, such as age, sex, BMI, ECOG PS, CCI and blood parameters were collected retrospectively from medical records. Information about neoadjuvant chemotherapy, cystectomy approach (open, laparoscopic or robotic) and urinary diversion (cutaneous ureterostomy, ileal conduit or neobladder) was also collected. We also reviewed histopathological data of resected specimens and recorded pathological diagnosis, pathological T stage, pathological lymph node metastasis and the presence of concurrent CIS.

### CT image analysis

**Figure 5:**
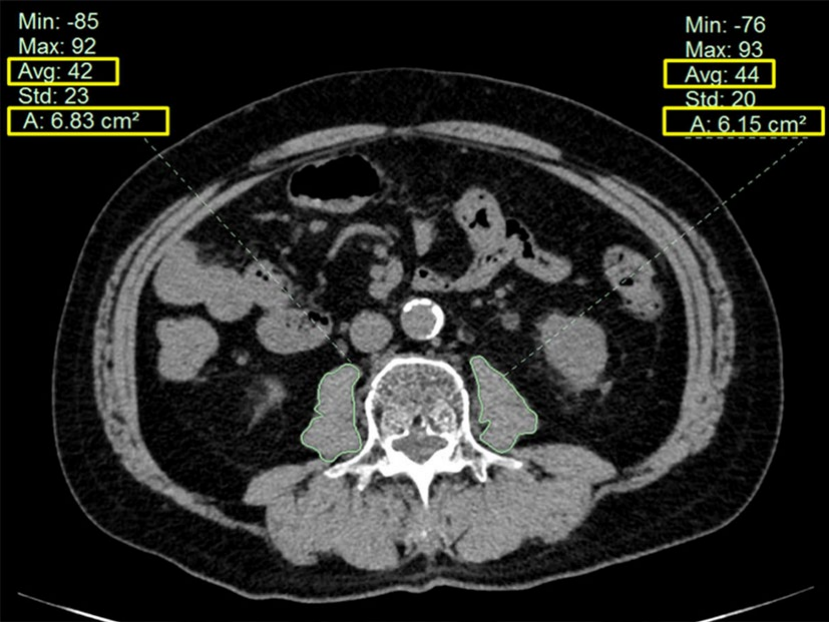
Measurement of area and density of each psoas muscle on the axial non-contrast computed tomography image at level L3.

Pre-surgical abdominal CT images were used for evaluation of total psoas muscle area and density. CT scans (5 mm collimation width) were performed using a GE LightSpeed 64-slice multidetector helical CT scanner (GE Healthcare Japan Corporation, Tokyo, Japan) and scanned images were analyzed on a GE workstation by one well-trained radiologist, blinded to patient outcomes, at each institution. A digital free-hand outline of the left and right psoas muscles was made on the axial non-contrast CT image at level L3 (Fig. 5). By this procedure, the area in cm^2^ and density in HU of each psoas muscle at this level were automatically calculated. To assess sarcopenia, PMI in cm^2^/m^2^ was calculated by normalizing the total psoas muscle area (left and right psoas muscle area) by the square of the patient’s height^7^. To assess myosteatosis, ATPD in HU was also calculated as an average of left and right psoas muscle density^20^.

### Statistical analysis

All statistical analyses were performed using JMP Pro 14. OS rate and CSS rate were determined by Kaplan–Meier method. Comparisons of OS and CSS between groups were performed using log rank tests. Comparison of patient demographics between groups were performed using chi-square tests, Fisher’s exact tests or Mann–Whitney U tests. Univariate and Multivariate Cox proportional regression analyses were performed to identify predictors of OS and CSS. In Cox proportional regression analyses, psoas muscle index and average total psoas density were analyzed as continuous variables. In all analyses, *P* < 0.05 was considered to be statistically significant.

## Data Availability

The datasets analyzed during the current study are available from the corresponding author on reasonable request.
